# A G→T splice site mutation of *CRYBA1/A3* associated with autosomal dominant suture cataracts in a Chinese family

**Published:** 2011-08-05

**Authors:** Zhenfei Yang, Qian Li, Zicheng Ma, Yuanyuan Guo, Siquan Zhu, Xu Ma

**Affiliations:** 1 Beijing Tongren Eye Center, Beijing Tongren Hospital, Capital Medical University, Beijing Ophthalmology & Visual Sciences Key Lab, Beijing, China; 2 National Research Institute for Family Planning, Beijing, China; 3 Peking Union Medical College, Beijing,China; 4 World Health Organization Collaborating Center for Research in Human Reproduction, Beijing, China

## Abstract

**Purpose:**

To identify the genetic defect in a five-generation Chinese family with congenital Y-suture cataracts.

**Methods:**

A five-generation Chinese family with inherited Y-suture cataract phenotype was recruited. Detailed family history and clinical data of the family were recorded. Candidate genes sequencing was performed to screen out the disease-causing mutation.

**Results:**

The congenital cataract phenotype of the family was identified as Y-suture cataract type by using slit-lamp photography. Direct sequencing revealed a G→T splice site mutation in crystallin, beta A1 (*CRYBA1/A3*).This mutation co-segregated with all affected individuals in the family and was not found in unaffected family members or 100 unrelated controls.

**Conclusions:**

Our study identified a novel type of a splice site mutation in *CRYBA1/A3* .The mutation was responsible for the congenital Y-suture cataracts in the family. This is the first report relating a G→T mutation of *CRYBA1/A3* to congenital Y-suture cataract.

## Introduction

Congenital cataracts, characterized by opacification of all or part of the eye's crystalline lens within the first year of life, are a leading cause of visual impairment or blindness in children [1]. The prevalence of congenital cataracts is 1 to 6 per 10,000 live births [2]. Cataracts can be isolated or occur in association with a large number of metabolic diseases and genetic syndromes. Congenital cataracts are most frequently inherited as autosomal dominant traits, but can also be inherited in an autosomal recessive or X-linked fashion [3]. According to morphology, congenital cataracts can be classified into several subtypes: whole lens, nuclear, lamellar, cortical, polar, sutural, pulverulent, cerulean, coralliform, and other minor subtypes [4].

Approximately half of all cataract families have crystallin mutations, including crystalline, alpha A (*CRYAA*), crystallin, alpha B (*CRYAB*), crystallin, beta A1 (*CRYBA1/A3*), crystallin, beta A4 (*CRYBA4*), crystallin, beta B1 (*CRYBB1*), crystallin, beta B2 (*CRYBB2*), crystallin, gamma C (*CRYGC*), crystallin, gamma D (*CRYGD*), crystallin, gamma S (*CRYGS*). About one quarter have connexin mutations in gap junctional proteins, including gap junction protein, alpha 3, 46kDa (*GJA3*), and gap junction protein, alpha 8, 50kDa (*GJA8*), with the remainder divided among the genes for heat shock transcription factor-4 (*HSF4*), aquaporin-0 (*AQP0*, *MIP*), and beaded filament structural protein-2 (*BFSP2*) [5].

We applied a functional candidate approach testing the known cataract-causing genes in a Chinese family. A G→T splice mutation in *CRYBA1/A3* was identified to be responsible for cataracts in the family. This is the first report to relate this mutation site to Y-suture cataracts also involving opacities of the nucleus.

## Methods

### Family data

A five-generation Chinese family from Shandong Province with a history of cataracts was recruited from Beijing Tongren Hospital, Capital Medical University, Beijing, China. The research was approved by the ethics committee of Capital Medical University. Informed consent was obtained from all participants of the family. The study protocol followed the principles of the Declaration of Helsinki.

Detailed family medical history was recorded by interviewing the family members. All participating members underwent ophthalmic examination, including visual acuity, slit-lamp examination, intraocular pressure measurement, ultrasonography, and fundus examination of the dilated pupil. Slit-lamp photography was performed to document the phenotype of the cataracts in the patients. One hundred unrelated subjects without cataracts were recruited from the Ophthalmology Clinic of Beijing Tongren Hospital as normal controls and were given complete ophthalmologic examinations. None of the controls exhibited eye diseases except mild myopia.

### Genomic DNA preparation

About 2 ml of peripheral blood was collected from the family members who took part in the study. Genomic DNA was extracted from blood using the QIAamp Blood kit (Qiagen, Valencia, CA).

### Mutation screening

We used the functional candidate gene analysis approach, including *CRYAA* (GenBank NM_000394), *CRYAB* (GenBank NM_001885), *CRYBA1* (GenBank NM_005208), *CRYBB1* (GenBank NM_001887), *CRYBB2* (GenBank NM_000496), *CRYGC* (GenBank NM_020989), *CRYGD* (GenBank NM_006891), *CRYGS* (GenBank NM_017541), *GJA3* (GenBank NM_021954), *GJA8* (GenBank NM_005267), *MIP* (GenBank NM_012064.3), *HSF4* (GenBank NM_001040667.2), and *BFSP2* (GenBank NM_003571). Each exon and intron-exon junction of the genes were amplified by polymerase chain reaction (PCR) using previously published primer sequences (Table 1) [6]. Each reaction mix (25 μl) contained 20 ng of genomic DNA, 1× PCR buffer,1.5 mM MgCl_2_, 0.2 mM dNTPs, 0.5 μM each of forward and reverse primers and 2.5 U of Taq DNA polymerase (Qiagen). A PCR program was performed for DNA amplifying: 95 °C for 5 min; followed by 35 cycles at 95 °C for 30 s, 57 °C-63 °C for 30 s (annealing temperature depending on different primer); 72 °C for 30 s; and a final extension at 72 °C for 10 min. The PCR products of the proband and one unaffected member were sequenced using an ABI3730 Automated Sequencer (PE Biosystems, Foster City, CA). The sequencing results were analyzed using Chromas 2.33 and compared with the reference sequence in the NCBI database. Then we screened the mutation in *CRYBA1/A3* from the sample of the family members and 100 ethnically matched controls to confirm the mutation.

**Table 1:** Primers used for PCR.

**Name**	**Forward (5′-3′)**	**Reverse (5′-3′)**
CRYAA-1	AGCAGCCTTCTTCATGAGC	CAAGACCAGAGTCCATCG
CRYAA-2	GGCAGGTGACCGAAGCATC	GAAGGCATGGTGCAGGTG
CRYAA-3	GCAGCTTCTCTGGCATGG	GGGAAGCAAAGGAAGACAGA
CRYAB-1	AACCCCTGACATCACCATTC	AAGGACTCTCCCGTCCTAGC
CRYAB-2	CCATCCCATTCCCTTACCTT	GCCTCCAAAGCTGATAGCAC
CRYAB-3	TCTCTCTGCCTCTTTCCTCA	CCTTGGAGCCCTCTAAATCA
CRYBA1–1	GGCAGAGGGAGAGCAGAGTG	CACTAGGCAGGAGAACTGGG
CRYBA1–2	AGTGAGCAGCAGAGCCAGAA	GGTCAGTCACTGCCTTATGG
CRYBA1–3	AAGCACAGAGTCAGACTGAAGT	CCCCTGTCTGAAGGGACCTG
CRYBA1–4	GTACAGCTCTACTGGGATTG	ACTGATGATAAATAGCATGAACG
CRYBA1–5	GAATGATAGCCATAGCACTAG	TACCGATACGTATGAAATCTGA
CRYBA1–6	CATCTCATACCATTGTGTTGAG	GCAAGGTCTCATGCTTGAGG
CRYBB1–1	CCCTGGCTGGGGTTGTTGA	TGCCTATCTGCCTGTCTGTTTCTC
CRYBB1–2	TAGCGGGGTAATGGAGGGTG	AGGATAAGAGTCTGGGGAGGTGG
CRYBB1–3	CCTGCACTGCTGGCTTTTATTTA	TCTCCAGAGCCCAGAACCATG
CRYBB1–4	CCAACTCCAAGGAAACAGGCATA	CCTCCCTACCCACCATCATCTC
CRYBB1–5	TAGACAGCAGTGGTCCCTGGAGA	AGCACTGGGAGACTGTGGAAGG
CRYBB1–6	CCTAGAAAAGGAAACCGAGGCC	AGCGAGGAAGTCACATCCCAGTA
CRYBB2–1	GTTTGGGGCCAGAGGGGAGTGGT	TGGGCTGGGGAGGGACTTTCAGTA
CRYBB2–2	CCTTCAGCATCCTTTGGGTTCTCT	GCAGTTCTAAAAGCTTCATCAGTC
CRYBB2–3	GTAGCCAGGATTCTGCCATAGGAA	GTGCCCTCTGGAGCATTTCATAGT
CRYBB2–4	GGCCCCCTCACCCATACTCA	CTTCCCTCCTGCCTCAACCTAATC
CRYBB2–5	CTTACCCTTGGGAAGTGGCAATGG	TCAAAGACCCACAGCAGACAAGTT
CRYGC-1	TGCATAAAATCCCCTTACCG	CCTCCCTGTAACCCACATTG
CRYGC-2	TGGTTGGACAAATTCTGGAAG	CCCACCCCATTCACTTCTTA
CRYGD-1	CAGCAGCCCTCCTGCTAT	GGGTCCTGACTTGAGGATGT
CRYGD-2	GCTTTTCTTCTCTTTTTATTTCTGG	AAGAAAGACACAAGCAAATCAGT
CRYGS-2	GAAACCATCAATAGCGTCTAAATG	TGAAAAGCGGGTAGGCTAAA
CRYGS-3	AATTAAGCCACCCAGCTCCT	GGGAGTACACAGTCCCCAGA
CRYGS-4	GACCTGCTGGTGATTTCCAT	CACTGTGGCGAGCACTGTAT
GJA3–1	CGGTGTTCATGAGCATTTTC	CTCTTCAGCTGCTCCTCCTC
GJA3–2	GAGGAGGAGCAGCTGAAGAG	AGCGGTGTGCGCATAGTAG
GJA3–3	TCGGGTTCCCACCCTACTAT	TATCTGCTGGTGGGAAGTGC
GJA8–1	CCGCGTTAGCAAAAACAGAT	CCTCCATGCGGACGTAGT
GJA8–2	GCAGATCATCTTCGTCTCCA	GGCCACAGACAACATGAACA
GJA8–3	CCACGGAGAAAACCATCTTC	GAGCGTAGGAAGGCAGTGTC
GJA8–4	TCGAGGAGAAGATCAGCACA	GGCTGCTGGCTTTGCTTAG
MIP-1	GTGAAGGGGTTAAGAGGC	GGAGTCAGGGCAATAGAG
MIP-2,3	CGGGGAAGTCTTGAGGAG	CACGCAGAAGGAAAGCAG
MIP-4	CCACTAAGG TGGCTGGAA	CTCATGCCCCAAAACTCA
HSF4–1	CATCCCATCCAGCCAGCCTTTTC	GGGCATGGGTGTTCACTGACGT
HSF4–2	CCTCGACCCATATCCCCGTAAG	GCAGGAGCAAGGCAGGCAGTC
HSF4–3	GCGGGAATGAGCAAAGAGGAGG	GCCAAGGCAGGAGAGAGGAAGG
HSF4–4	TCCCCAGCCTCGCCATTCT	CCCGGTGAAGGAGTTTCCAGAG
HSF4–5	GCTGGGGCCTGAGGGAG	GGCTTCCATCTTCTCTTCCTTTT
BFSP2 (1a)	AATGCACAAACCCAAATGGT	AGGCCCTGSSGACACT
BFSP2 (1b)	GAGAGGCGAGTGGTAGTGGA	GGCCTCAGCCTACTCACAAC
BFSP2 (2)	TGCAGACAGAGCATTTCCAC	GAGGGGTGTGAGCTGGATAA
BFSP2 (3)	GCTGCAATTGCCTTCATTTT	GGGTAACCTGACCCAACTTCA
BFSP2 (4)	TCTGTGAAGCCTGTGTCTGG	CCCGGCCTCAATTATTCTTT
BFSP2 (5)	ACCCAGGAGGAGGAGGTTGT	GGGAATCCCCTGGAAACTAA
BFSP2 (6)	GGGGAATAGTCCAGGCTACC	ATGGGTGCCTATGTGAGAGGG
BFSP2 (7)	TTGTTCCAAAGGCCAGATTC	CACTCAAGGGAATCCTTCCA

## Results

### Clinical evaluation

**Figure 1:**
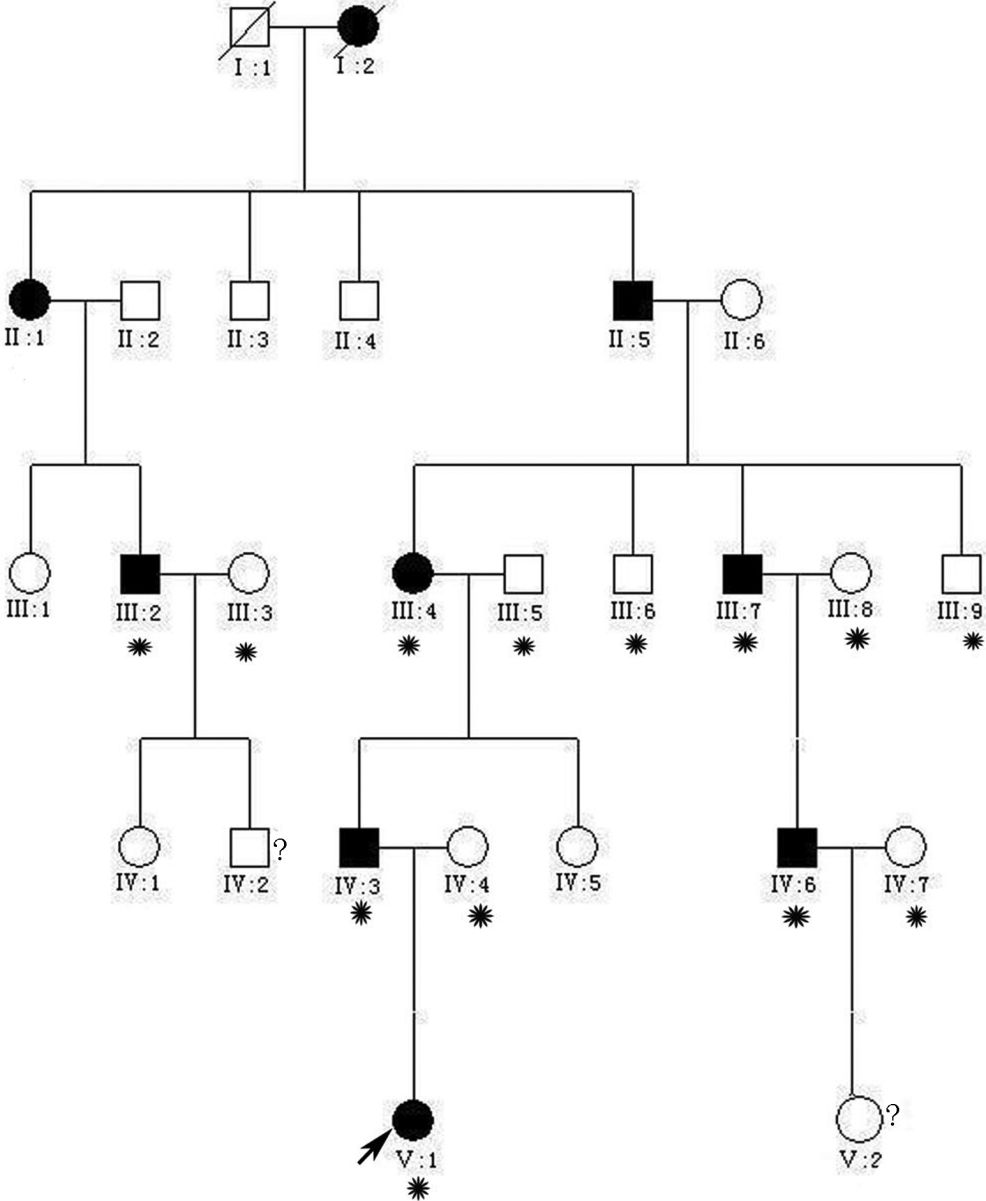
A five-generation Chinese family with autosomal dominant cataract. The black symbols indicate individuals with a diagnosis of congenital cataracts by doctors. The arrow indicates the proband. The asterisks indicate family members who attend this study. Family members IV:2 and V:2 were only several months old and did not take part in the study. We do not know whether they are affected.

**Figure 2:**
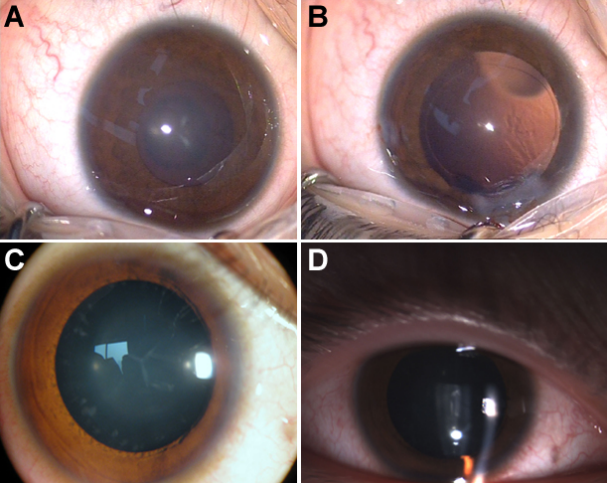
Slit lamp photographs of different individuals. Slit lamp photographs of individual V:1 (**A** and **B**). **A**: Y-suture opacities of the lens involving the nucleus. **B**: Slit lamp photograph of the eye after the lens was extracted. **C** and **D**: The photographs of individual IV:6 show Y-suture opacities of the lens involving the nucleus and peripheral cortex. The phenotypes of both are almost the same.

Thirteen family members of a five-generation Chinese family with a history of cataracts participated in the study (six affected and seven unaffected individuals; Figure 1). All patients in this family had bilateral cataracts. Most patients experienced decreased visual acuity at 3–4 years old, and then their visual acuity decreased gradually until surgery was required. The proband, who was a 3-year-old girl, experienced a decrease in vision at 1.5 years old and had been diagnosed with bilateral cataracts at age 3. Slit-lamp examination revealed opacification of Y- sutue cataracts with opacities involving nucleus. The girl’s best corrected visual acuity was 0.3/0.3. Her clinical features were similar to those of her uncle (IV:6) with peripheral cortical opacity (Figure 2). His best corrected visual acuity was 0.3 /0.4. The affected member IV:3, who was the father of the proband, had undergone cataract removal at age 8.

### Mutation analysis

**Figure 3:**
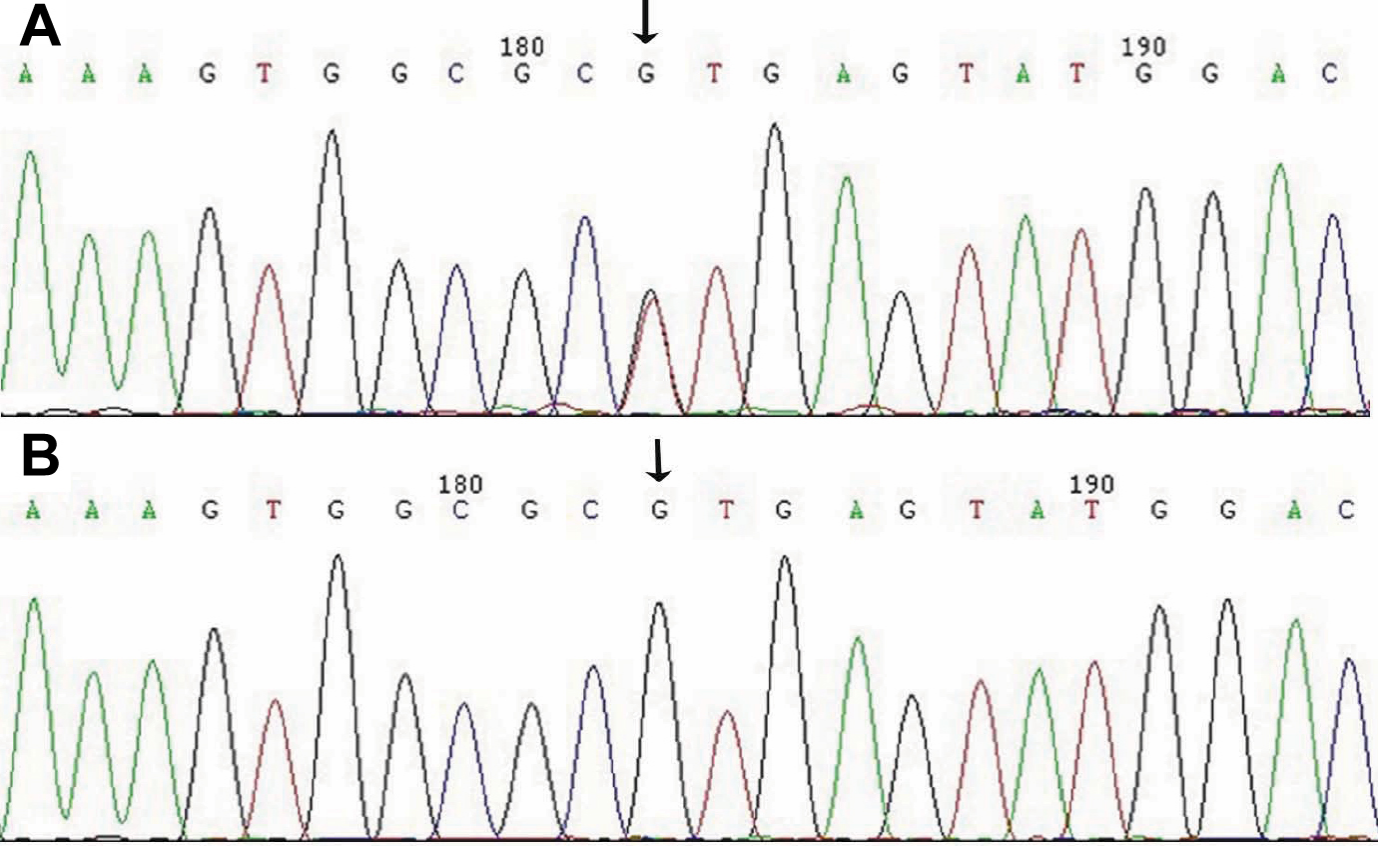
Sequence analysis of *CRYBA1/A3* at exon 3. **A**: Sequence of affected (individual V:1). **B**: Sequence of unaffected individual (individual IV:5). In panel **A**, the mutation G→T was evident at the first base of intron 3, which was identified in all patients of the family, but was not found in the unaffected family members nor in the 100 unrelated control subjects.

Through direct gene sequencing of the coding regions of the candidate genes, we identified an IVS3+1 G→T substitution in the donor splice site of intron 3 in *CRYBA1/A3* in all affected individuals (Figure 3). However, we did not find this mutation in any unaffected family members or in the 100 unrelated controls. We did not find any other mutations in this family except for a few non-pathogenic single nucleotide polymorphisms (SNPs).

## Discussion

In this study we identified a splice site mutation of *CRYBA1/A3* in a five-generation Chinese family with Y-suture opacities of the lens involving embryonic and fetal nuclei.

Sutural cataracts affect the sutural regions of the nucleus, at which the ends of the lens fiber cells meet. Sutural cataracts may occur in isolation or be associated with opacities involving other lens regions. There is some correlation between the pattern of expression of the mutant gene and the morphology of the resulting cataract.

To date, seven genes have been identified to be associated with suture cataracts, including *BFSP2, CRYBA1/A3, CRYBBA, CRYBB2, GJA8, FTL, CRYGA.* Among these genes, almost all the mutations of *BFSP2* are associated with suture cataract phenotype. *CRYBA1/A3* has great correlation with suture cataracts (Table 2).

**Table 2:** Summary of mutations responsible for suture cataract.

**Gene**	**Position**	**Sequence change**	**Lens phenotype**	**Reference**
*CRYGA*	2q33-q35	Unknown	Sutural cataract	[7]
*FTL*	19q13.3	32 G>A	Y-suture congenital cataract	[8]
*GJA8*	1q21	235G>C	Full moon with Y-suture cataract	[9]
*GJA8*	1q21	262C>A	Y-suture cataract	[10]
*BFSP2*	3q21.3-q27.2	697–699delGAA	Y-suture cataract	[11]
*BFSP2*	3q21.3-q27.2	697–699delGAA*	Congenital nuclear and sutural cataract	[12]
*BFSP2*	3q21.3-q27.2	696–698delGAA	Progressive sutural congenital cataract	[13]
*BFSP2*	3q21.3-q27.2	696–698delGAA	Progressive congenital cataract with suture and cortex opacity	[14]
*CRYBA1*	14q13-q21	IVS3+1G>A	Sutural, nuclear, and peripheral cortical opacity	[15]
*CRYBA1*	4q13-q21	IVS3+1G>C	Zonular and sutural cataract	[16]
*CRYBA1*	4q13-q21		Y-shaped sutural cataract	[17]
*CRYBA1*	4q13-q21	IVS3+1 G>A	Progressive childhood cataract with Y-suture opacity	[18]
*CRYBB2*	22q11.23	483C>T	opacities with suture and cerulean	[19]
*CRYBB1*	22q12.1	658G>T	Ustlike cataract with the anterior and posterior Y-suture opacities	[20]

So far, in the *CRYBA1/A3* gene, three types of mutations have been associated with autosomal dominant cataracts. Our report of IVS3+1 G→T will be the fourth type of *CRYBA1/A3* mutation. The first one is the IVS3+1 G→A mutation. Regarding IVS3+1 G→A, in 1998 Kannabiran et al. [21] reported an Indian family with zonular cataracts with sutural opacities. In 2008, Devi et al. [22] reported another two Indian families with zonular lamellar cataracts. In 2004, Burdon et al. [15] reported an Australian family with Y-sutural cataracts. In 2010, Gu et al. [23] identified a Chinese family with posterior polar cataracts, which was the first time this mutation was found in the Chinese population. Also in 2010, Zhu et al. [18]reported a Chinese family with progressive childhood cataracts characterized by opacities in the fetal nucleus and peripheral cortex. The second type of mutation is IVS3+1 G→C. In 2000, Bateman et al. [16] reported a Brazilian family with varied clinical characteristics among the affected members. The affected individuals who were examined had pulverulent opacities in the embryonal nucleus and sutures and star-shaped, shieldlike, or radial opacities in the posterior embryonal nucleus. The third type of mutation is a 3-bp deletion at positions 276–281 in exon 4, which causes an in-frame deletion of a glycine residue at position 91 (ΔG91). In 2004, Qi et al. [24] identified a Chinese family with nuclear cataracts. In 2007, Lu et al. [25] reported two Chinese families with pulverulent congenital cataracts (Table 3).

**Table 3:** Summary of mutations in *CRYBA1/A3* responsible for congenital cataract.

**Exon**	**Nucleotide**	**Amino acid**	**Phenotype**	**Reference**
IVS3	IVS3+1G>A	Splice site mutation	Zonular cataract with sutural opacity	[21]
IVS3	IVS3+1G>A	Splice site mutation	Zonular lamellar cataract	[22]
IVS3	IVS3+1G>A	Splice site mutation	Y-sutural,mild nucleus and cortical dot cataract	[15]
IVS3	IVS3+1G>A	Splice site mutation	Posterior polar cataract	[23]
IVS3	IVS3+1G>A	Splice site mutation	Progressive childhood nucleus and peripheral cortex cataract	[18]
IVS3	IVS3+1G>C	Splice site mutation	Pulverulent, star-shaped, shieldlike and radial cataract	[16]
EX4	278–280delGGA	P.91Glydel	Nuclear cataract	[24]
EX4	279–281delGGA276–278delGGA	P.91Glydel P.91Glydel	Pulverulent congenital cataracts	[25]
EX4	279–281delGGA	P.91Glydel	Congenital nuclear cataract	[26]

*CRYBA1/A3* consists of six exons encoding two proteins (βA3-crystallin and βA1-crystallin) by using an alternative translation initiation site. βA1/A3-crystallin consists of seven protein regions: four homologous (Greek key) motifs, a connecting peptide, and NH2- and COOH-terminal extensions.

In the *CRYBA1/A3* gene, the first two exons encode the sequence of the N-terminal arm, and exons 3–6 encode the Greek key motifs 1–4 [27]. The G at position +1 of the 5′ (donor) splice site is highly conserved, and mutation of this base can be expected to disrupt the splice site [28]. In this study the mutation at IVS3+1 G→T can be expected to skip the donor splice junction, which may cause the wrong junction of the exons in *CRYBA1/A3.* This may result in premature termination of the polypeptide. In this condition, it would cause structural instability and disrupt the folding of the protein [21].

In conclusion, we have identified a new type IVS3+1 G→T mutation of the *CRYBA1/A3* gene associated with Y-sutural congenital cataracts in a Chinese family. This mutation supports the role of the *CRYBA1/A3* gene in human cataract formation and provides more evidence of genetic heterogeneity of congenital cataracts.
